# Numerical analysis of propeller induced ground vortices by actuator disk model

**DOI:** 10.1007/s12650-017-0444-4

**Published:** 2017-09-01

**Authors:** Y. Yang, L. L. M. Veldhuis, G. Eitelberg

**Affiliations:** TU Delft, 2629HS Delft, Netherlands

**Keywords:** Ground vortex, Propeller aerodynamics, Large eddy simulation, Actuator disk, Vorticity source

## Abstract

**Abstract:**

During the ground operation of aircraft, the interaction between the propulsor-induced flow field and the ground may lead to the generation of ground vortices. Utilizing numerical approaches, the source of vorticity entering ground vortices is investigated. The results show that the production of wall-parallel components of vorticity has a strong contribution from the wall-parallel components of the pressure gradient on the wall, which is generated by the action of the propulsor. This mechanism is a supplementation for the vorticity transported from the far-field boundary layer, which has been assumed the main vorticity source in a number of previous publications. Furthermore, the quantitative prediction of the occurrence of ground vortices is performed from the numerical results. As the distance of the propeller form the ground decreases, and as the thrust of the propeller increases, ground vortices are generated from the ground and enter the propeller. In addition, the vortices which exist near the ground but does not enter the propeller plane are observed and visualized by three-dimensional data.

**Graphical abstract:**

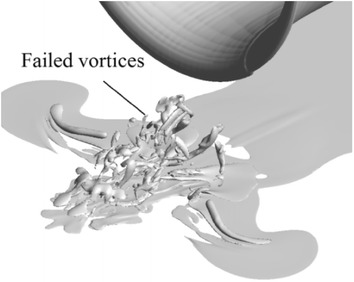

## Introduction

A system of ground vortices can be observed from the ground toward the engine during aircraft ground operations when rain droplets are present or air condensation occurs in the vortex region. The ground vortex impingement on the blades can lead to the dynamic loading, structural vibration (Coton et al. 2005), noise generation (Povinelli et al. 1972) as well as foreign object damage (Rodert and Garrett 1955). The problem of ground vortices ingested by engines is well known in the aerospace community and justifies numerous investigations over the past 60 years.

Extensive research on the detail of ground vortices have been conducted on simplified turbofan models, e.g. suction tubes (De Siervi et al. 1982; Murphy and MacManus 2011; Wang and Gursul 2012; Trapp and Girardi 2010). These investigations have greatly improved the understanding on the formation mechanism of ground vortices. For the headwind condition, which is the situation of our investigation, ground vortices are proposed to be due to the ingestion of boundary layer (De Siervi et al. 1982; Bissinger and Braun 1974; Murphy et al. 2010). For the crosswind condition, the vortex that is shed from the shroud of the engine is proposed to be the ground vortex origin (De Siervi et al. 1982).

During the study of ground vortices in the wind tunnel, the wind-tunnel wall function as the ground and it is inevitable that there is a boundary layer above the wall. Therefore, it was concluded that the vorticity source for the ground vortices was solely the approaching boundary layer. However, when there is no far-field boundary layer, e.g. when the aircraft taxis on the runway or it is stationary, the vorticity should be generated locally by the engine suction on the ground. Fundamental studies (Lighthill 1963; Morton 1984; Hornung 1989; Wu and Wu 1993) showed that the vorticity generating mechanism on the wall involves the tangential pressure gradient and external acceleration of the wall. The area influenced by the engine suction on the ground has a pressure gradient that certainly feeds the ground vortex with vorticity. Therefore, one objective of the paper is to confirm the vorticity source from pressure gradient on the ground in addition to the vorticity transported from the far-field boundary layer.

Previous investigations showed that the occurrence of ground vortices, which were induced by turbofans, depended on the engine height above the ground and the engine suction strength (Murphy 2008). However, such investigations have not been performed on propellers yet. Therefore, the other objective of the paper is to determine the occurrence of ground vortices induced by a propeller.

## Numerical methods

### Actuator disk model

For the study of ground vortices induced by a propeller, the actuator disk model is selected. The actuator disk model adds an axial momentum in the flow at the position of the propeller disk plane. The reasons to choose this model are as follows. First, the ground vortices are assumed to be mostly determined by the axial force (thrust) of the propeller and the torque of the propeller has a negligible influence, because ground vortices are mostly located upstream of the propulsor (Murphy and MacManus 2011; Wang and Gursul 2012; Murphy et al. 2010) and the induced velocities by the torque are negligible upstream of the propeller (Weick 1930). Second, the actuator disk model can easily be implemented in the CFD code. The limitation of the actuator disk model is the exclusion of the blade passing effect. However, as found in (Yang et al. 2016), although the blade passing effect is significant in the region close to the blades, the effect on the flow field near the ground is negligible. Therefore, it is assumed that the flow field near the ground induced by the actuator disk model represents the real situation with sufficient accuracy for the purposes of this study.

**Fig. 1:**
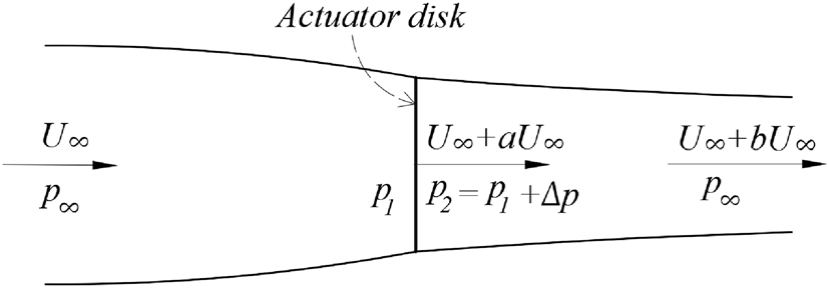
Schematic of the actuator disk model

The actuator disk theory is also known as the one-dimensional momentum theory, and it is the oldest mathematical model for propellers (Froude 1889). It was first applied on marine propellers by Rankine (Rankine 1865) and developed by Froude (Froude 1878). The idea of momentum theory is that the momenta of flow far upstream and far downstream of the propeller are not equal, the difference of which is the momentum added to the flow by the propeller. To explain this model, the propeller is considered as a stationary disk in a moving fluid. This conceptual model for the flow going through the propeller is shown in Fig. 1.

The sum of streamlines going through the disk from far upstream to far downstream positions define the stream tube. The flow is highlighted at three planes in the stream tube, the plane far upstream, the plane of the actuator disk, and the plane far downstream. The plane far upstream of the propeller has the velocity of $U_{\infty }$ and pressure $p_{\infty }$, which are the free stream velocity and the ambient pressure, respectively. The pressure decreases to $p_{1}$ and the velocity increases to $U_{\infty } + aU_{\infty }$ at the front surface of the disk, where *a* is the coefficient of the increment of the axial velocity at the disk. After passing through the disk, the pressure has an increment $\Delta p$ and the velocity remains $U_{\infty } + aU_{\infty }$ due to the mass conservation. At the plane far downstream, the pressure decreases to the ambient pressure $p_{\infty }$, and the velocity increases to $U_{\infty } + bU_{\infty }$, where *b* is the coefficient of the increment of the axial velocity in the far downstream flow.

The thrust generated by the propeller (*T*) can be determined by the pressure jump multiplied by the area of the disk, and it is equal to the rate of change of axial momentum between the planes far upstream and far downstream.

1 $$T = \Delta p \cdot S_{\text{disk}} = \rho S_{\text{disk}} (U_{\infty } + aU_{\infty } )bU_{\infty } .$$

$S_{\text{disk}}$ is the area of the actuator disk.

The thrust as shown in Eq. (1) is normalized as

2 $$T_{\text{c}} = T/(\rho U_{\infty }^{2} D^{2} ),$$

where *D* is the diameter of the propeller. $T_{\text{c}}$ is termed as the thrust coefficient of the propeller.

The coefficient of the axial velocity increment *b* is related to the thrust coefficient of the propeller, and the detail is elaborated in many propeller textbooks, e.g. (Weick 1930),

3 $$b = - 1 + \sqrt {1 + \frac{8}{\pi }T_{\text{c}} } .$$

From Eq. (3), it is found that when the thrust coefficient of the propeller increases, the axial velocity in the slipstream of the propeller increases. Therefore, the diameter of the stream tube downstream of the propeller decreases. At the same time, the diameter of the stream tube upstream of the propeller increases, because the mass flow rate of the propeller increases.

The equivalent velocity in the slipstream of the actuator disk model, which is utilized for the normalization of ground vortices in the following context, is termed as $U_{\text{eq}}$,

4 $$U_{\text{eq}} = \left( {1 + b} \right)U_{\infty } .$$

### Setup of simulations

To enable a detailed analysis of the propeller induced flow field, a numerical study is performed which is based on large eddy simulations (LES). LES is a numerical approach to divide the variables (e.g. velocities) into resolved and unresolved parts. The resolved parts or large-scale quantities that are computed directly. The unresolved or sub-grid scale quantities are modelled, such as the dynamic Smagorinsky–Lilly subgrid model adopted in this paper. A detailed description of the LES method is given in Pope (2000). The main advantage of choosing LES for the simulation of ground vortices is that it is capable of capturing the unsteady effects much better than an unsteady Reynolds-averaged Navier–Stokes (uRANS) approach, and does not require such extensive computational power as direct numerical simulation (DNS) (Chumakov 2005). In addition, the ground vortices which are induced by a suction tube model using the LES approach are consistent with the experimental results as reported in (Trapp and Girardi 2010).

**Fig. 2:**
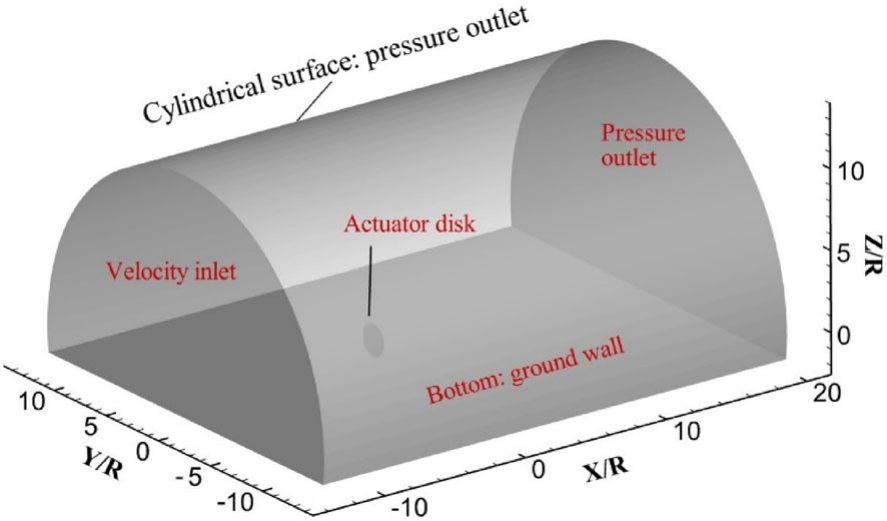
Overview of the computational domain and boundary conditions. Front surface: velocity inlet; cylindrical surface: pressure outlet; disk: actuator disk; bottom surface: ground wall; back surface: pressure outlet

The computational domain is a semi-cylindrical volume where the actuator disk model is inserted, as shown in Fig. 2. The origin of the coordinate system is at the centre of the actuator disk, and the *X*-axis is along the axial direction of the disk. Other axes follow the definition of the right-handed Cartesian coordinate system. The dimension of the domain is: $[ - 13.1 R,20.0 R]$ in the *X* direction, and $[ - 13.1 R,13.1 R]$ in the *Y* direction on the ground plane, where *R* is the radius of the propeller. The diameter, *D*, and the distance of the actuator disk model above the ground, *h*, are according to the experimental test conducted on a propeller model (Yang et al. 2014). A brief description of the experimental setup is shown in Fig. 4. The front and back boundaries of the semi-cylinder are prescribed as velocity inlet and pressure outlet boundary conditions, respectively. The cylindrical surface of the semi-cylinder as shown in Fig. 2, is an outlet boundary condition as well, which is consistent with the flow condition in an open jet facility.

The velocity profile that is prescribed at the position of the velocity inlet is available from experimental data (Yang et al. 2016), and the free-stream velocity is $U_{\infty } = 2.7 \,\,{\text{m/s}}$. The fluctuation velocity at the velocity-inlet boundary is modelled by the vortex method, which is applied by adding “vortex points” on the velocity inlet via a fluctuating vorticity field. The magnitude of each vortex point is determined by the turbulence intensity and its distribution is assumed a Gaussian like profile [the details of this vortex points method are reported in Mathey et al. (2006)]. The turbulence intensity is 0.5% which is determined from the experimental measurement. No-slip wall boundary condition is prescribed on the ground. The pressure jump prescribed on the actuator disk model is set according to the thrust of the propeller as shown in Eq. (1).

**Fig. 3:**
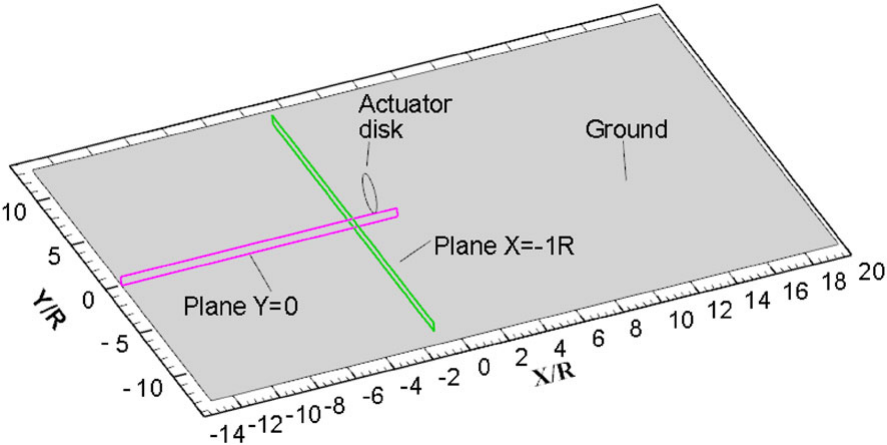
Definitions of planes for analysis of vorticity source, which will be performed in Sect. 4 of the paper. Plane $Y = 0$ is utilized to analyse the production of the *Y*-component vorticity, and plane $X = - 1 R$ is utilized to analyse the production of the *X*-component vorticity

Two planes are defined in Fig. 3 for the analysis of vorticity source near the ground, which will be performed in Sect. 4 of the paper. One is part of the symmetry plane $Y = 0$, and the other one is part of the plane $X = - 1R$. These two planes near the ground are selected because the vorticity source of ground vortices is believed to be originated from the ground.

**Fig. 4:**
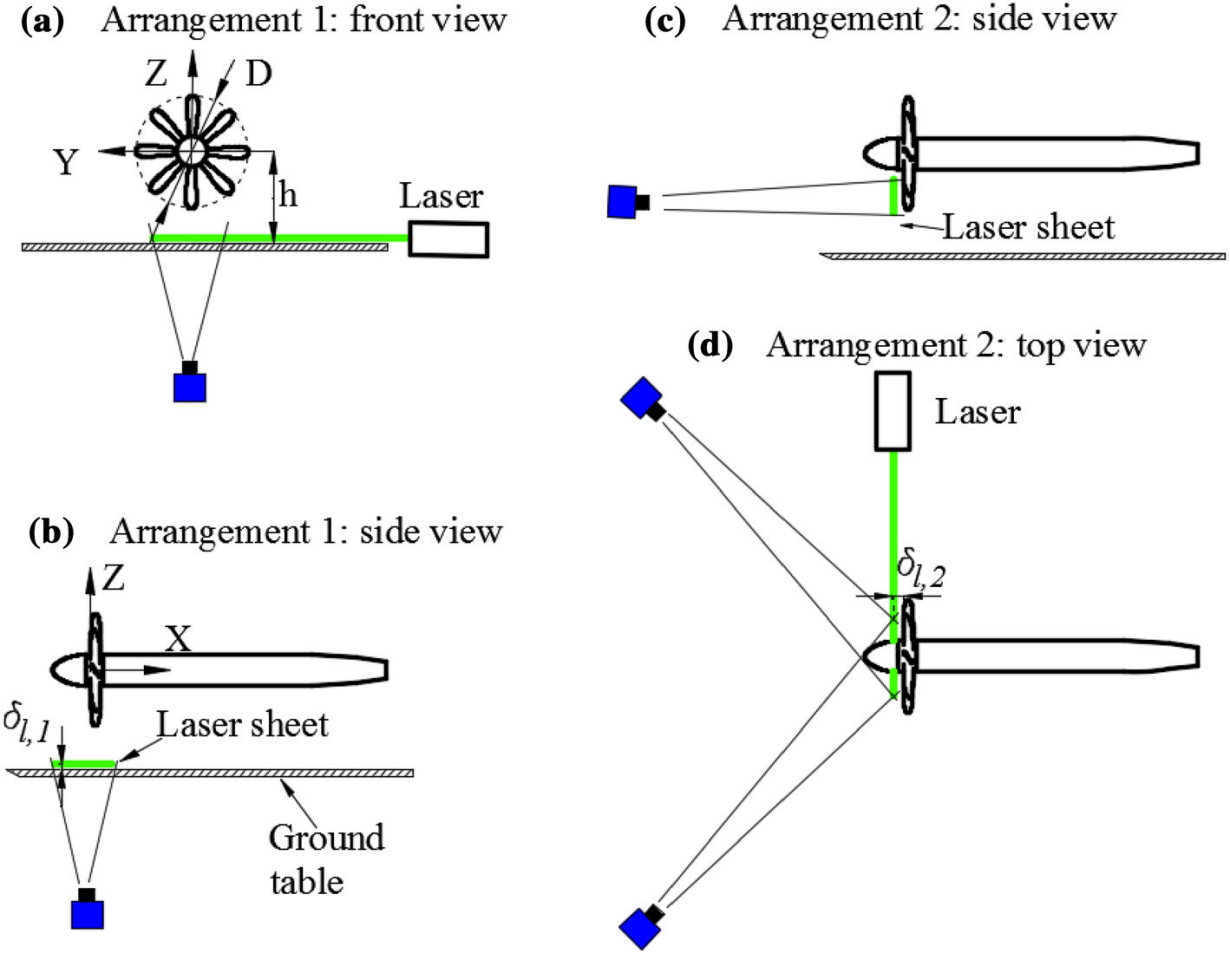
PIV arrangement 1 for the measurement in the wall parallel plane and PIV arrangement 2 for the measurement in the plane upstream of propeller. **a** Front view of PIV arrangement 1; **b** side view of PIV arrangement 1; **c** side view of PIV arrangement 2; **d** top view of PIV arrangement 2

To help understand the setup of the numerical simulations, which is intended to be the same as the experiment (Yang et al. 2014), the arrangements of the experiment are shown in Fig. 4. A flat table is positioned under the propeller to represent the ground operation of the propeller. There are two particle image velocimetry (PIV) arrangements to analyse the flow field involving ground vortices. For PIV arrangement 1, measurements are conducted at $\delta_{l,1}$ = 0.046 *R*, which is 7 mm above the ground, as shown in Fig. 4a, b. For PIV arrangement 2, measurements are carried out at $\delta_{l,2} = 0.08 R$, which is 12 mm upstream of the leading edge of the propeller blade, as shown in Fig. 4c, d. In addition, the coordinate system is defined in Fig. 4, which is the same as numerical simulations. The flow fields in these two PIV measurement planes are compared with that from numerical simulations at the same position and shown in Sect. 3.

**Fig. 5:**
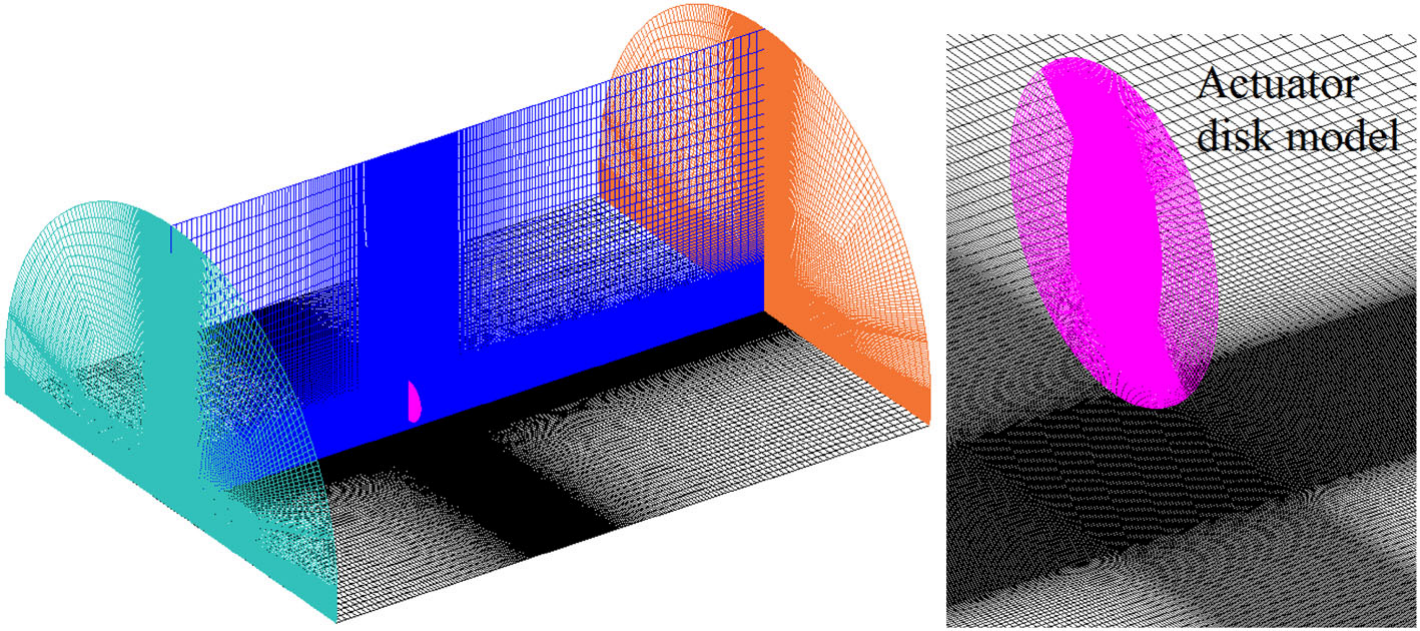
*Left* structured mesh of the computational domain; *right* detail of the mesh on the disk and below the disk

**Fig. 6:**
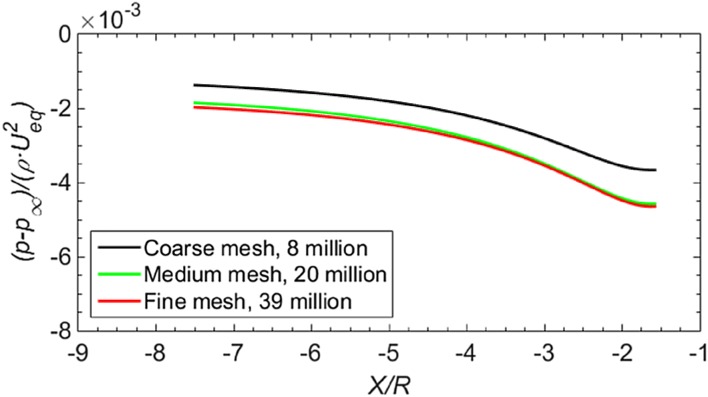
Study of mesh convergence by investigating the pressure distribution on the ground along the symmetry line

The structured mesh is generated by ANSYS ICEM and shown in Fig. 5. The thickness of the first layer inside the wall boundary achieves $Y^{ + } = 1$, which is based on the reference distance from the inlet to the actuator disk. The final node number is determined by evaluating the convergence of pressure distribution on the ground, the gradient of which is required for the source of ground vortices. The pressure distribution ($p - p_{\infty }$) on the symmetry line on the ground is shown for three different grid sizes in Fig. 6. This pressure is normalized by $\rho U_{\text{eq}}^{2}$, which is related to the thrust generated by the propeller. Because the pressure distribution on the ground is determined by the thrust generated by the propeller, such a normalization scales the pressure on the ground with the propeller loading. The pressure value for 20 million nodes is approximately 2% lower than that at the node number of 39 million, and the mesh is, therefore, considered converged at the node number of 20 million.

The commercial software ANSYS FLUENT is used for the parallel computation. It runs on a cluster with 48 processors. FLUENT uses a control volume-based technique to solve the control equations numerically. The pressure-based solver is adopted because the flow domain is incompressible (based on the velocity in the slipstream of the actuator disk model). Spatial discretization uses the bounded central-differencing scheme which is a second-order-accurate central differencing method. Velocity and pressure coupling is achieved by the SIMPLE algorithm in FLUENT (ANSYS 2006). The temporal discretization of the transient derivatives uses the second-order implicit schemes. The time step is set at a constant value of 0.0005 s which is determined by Courant–Friedrichs–Lewy (CFL) condition (${\text{CFL}} = 0.9$) and the frequency of the flow field [Strouhal number of the ground vortices induced by a suction tube model (Wang and Gursul 2012; Yang et al. 2016)].

The computation starts from the steady calculation for 3000 steps until the residuals of the pressure and velocities converge. The calculation is then switched to LES for 2 s, which corresponds to a time of 138 $R/U_{\text{eq}}$. The calculation continues for another 1 s which corresponds to a time of 69 $R/U_{\text{eq}}$ and it is utilized for sample averaging.

## Example flow fields

**Fig. 7:**
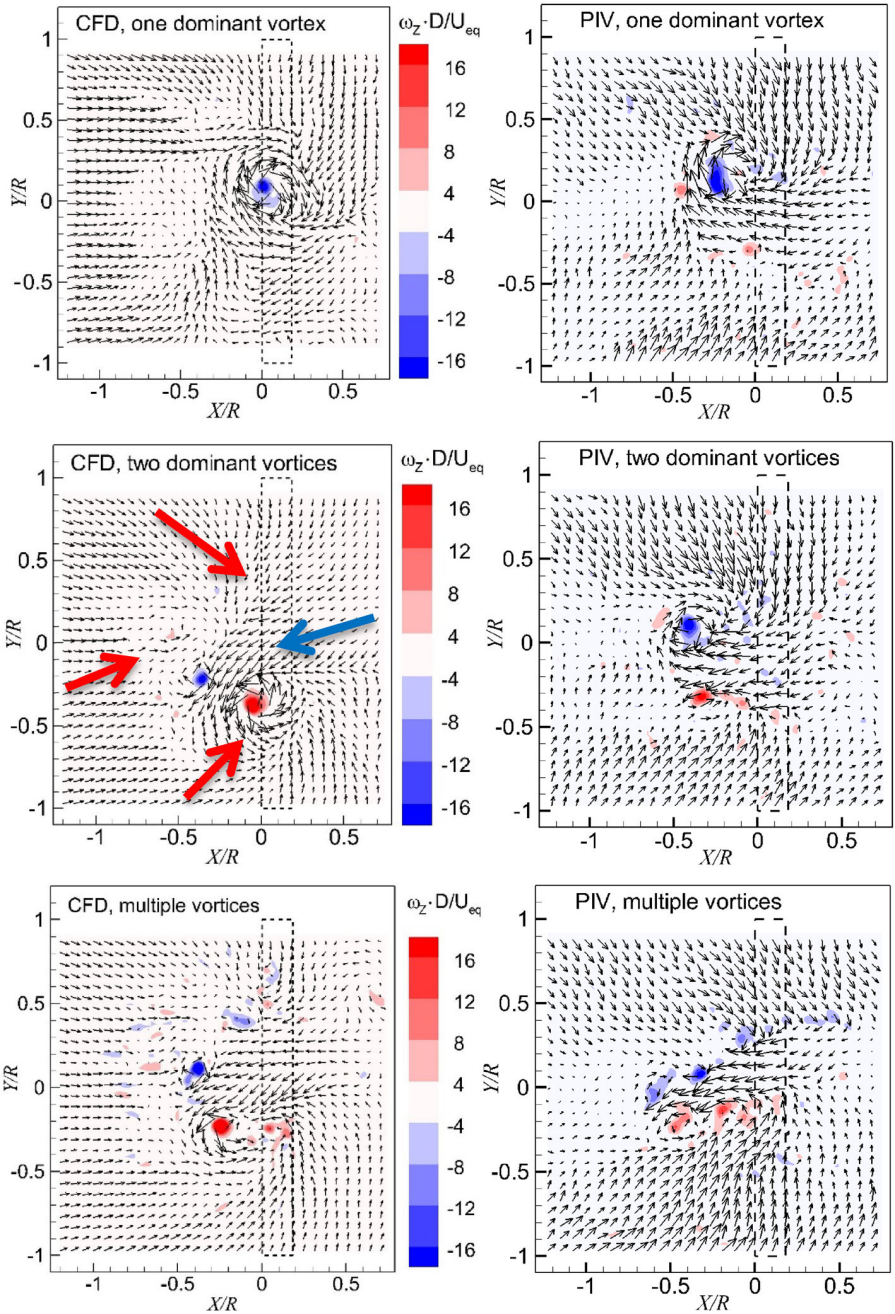
Instantaneous flow fields above the ground ($T_{\text{c}} = 27.3$, $h/R = 1.46$). *Top* one dominant vortex; *middle* two dominant vortices; *bottom* multiple dominant vortices. The *dashed lines* show the location of the propeller. The *red arrows* are indicators of the flow moving downstream, and the *blue arrow* is that of the flow moving upstream

Typical instantaneous flow fields in the wall-parallel plane, which is 7 mm (0.046*R*) above the ground as show in Fig. 4, are presented in Fig. 7. The height ratio of the propeller is set at $h/R = 1.46$. The thrust coefficient of the propeller is set at $T_{\text{c}} = 27.3$. The velocity in the slipstream of the propeller as defined in Eq. (4) is $U_{\text{eq}} = 22.7\,\, {\text{m/s}}$.

The figures on the left-hand side are from the CFD simulations and the figures on the right-hand side are from PIV measurements [the PIV data are reported in (Yang et al. 2014)]. These instantaneous flow fields, which are selected at random instants, are intended to show different topologies near the ground, specifically the different number of dominant vortices in the flow fields. The evolution of flow fields in the time domain is discussed in detail in Fig. 10 by 3D data, and in (Yang et al. 2016) which was based on high frequency PIV data.

The free stream direction is from the left to the right, and the projection of the propeller on the measurement plane is represented by the dashed rectangle. The figures are colour-coded with non-dimensional wall-normal component of vorticity. Because $U_{\text{eq}} /D$ is related to the averaged thrust of the propeller in the slipstream, normalizing the vorticity inserted into the inflow in the chosen way scales the inserted vorticity with the vorticity generated by the propeller.

The flow upstream of the propeller is dominated by the flow in the downstream direction (marked by red arrows in the middle row of Fig. 7), while a flow in the upstream direction (blue arrow) can be observed in the region underneath the propeller projection, which is mainly due to the suction effect of the propeller. This non-uniform flow near the ground induces a gradient in the streamwise velocity field, i.e. $\partial U_{X} /\partial Y$. Both PIV measurement and CFD results show similar flow topologies in the same plane close to the wall.

The location of vortices is identified by the local maximum of vorticity magnitude, as well as the visualization of the flow field. The maximum vorticity magnitude, which is selected for the detection of the vortex location in our investigation, is also extensively adopted in literature during detecting ground vortices (Wang and Gursul 2012; Murphy et al. 2010). However, shear layers can be recognized as vortices by such a method. Therefore, the visualization of the flow field is also utilized as a supplementation method. There is one dominant peak of vorticity as shown in the top row of Fig. 7, and there is one dominant iso-surface of the vorticity magnitude which is not shown here, therefore, the flow field is interpreted to have one dominant ground vortex. In a similar manner, the flow field in the middle row of Fig. 7 is interpreted to have two dominant ground vortices, and the flow field in the bottom row of Fig. 7 is interpreted to have multiple vortices.

**Fig. 8:**
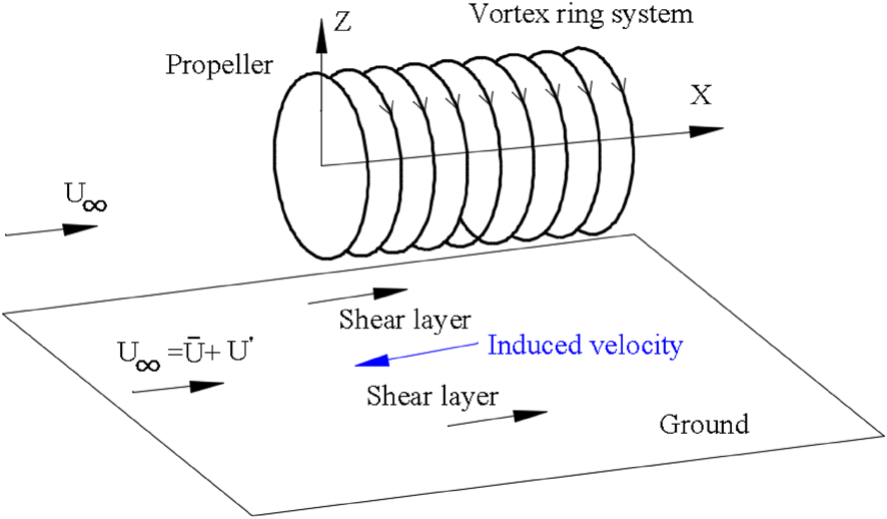
Two factors assumed to account for the unsteadiness of ground vortices: fluctuation of free stream $U^{'}$ and shear layers near the ground induced by the vortex system in the propeller slipstream

Variation of the number of ground vortices was reported in Brix et al. (2000) as well, where a suction tube model was used. The reason given in Brix et al. (2000) stated that the vertical vortex lines (i.e. the velocity gradient in the lateral direction of the free stream) played a significant role in this process. Due to the fluctuation of the free stream, the velocity gradient in the lateral direction varies in the time domain, and the incoming vertical vortex lines become unstable. When the free stream velocity is set to be zero, the ground vortices (a pair of vortices) become stable again. This explanation also describes our situation. The free-stream velocity is decomposed into mean and the fluctuation parts, $\bar{U}$ and $U^{'}$, respectively, as shown in Fig. 8. The fluctuation velocity $U^{'}$ contributes to the variation of the number of ground vortices.

In our case, one more source of vertical vortex line(s) is in the flow downstream of the propeller near the ground, which is due to the effect of the propeller slipstream. However, it should be noted this slipstream effect is not involved in the turbofan, which has a shroud. These vertical vortex lines are ascribed to the vortex system in the propeller slipstream, which can be decomposed into vortex rings and longitudinal vortex lines (Conway 1998) (the effects of longitudinal vortex lines follow the same trend and only the vortex rings are considered here). The effect of the vortex rings induces flow moving toward the upstream direction, as shown in Fig. 8. This upstream flow and the free stream generate shear layers involving vertical vortex lines. These vortex lines are transported to the region where ground vortices occur. Due to the turbulent feature of the vortex system in the propeller slipstream, these vertical vortex lines become unstable, just like the ground vortices. This vortex ring system also exists in the CFD simulations, which is generated by the shear layer between the slipstream and the free stream (as shown in Fig. 10), hence the CFD simulations can simulate this effect qualitatively as well. Furthermore, the source of unstable vertical vortex lines could also be due to the recirculation of the flow near the ground.

**Fig. 9:**
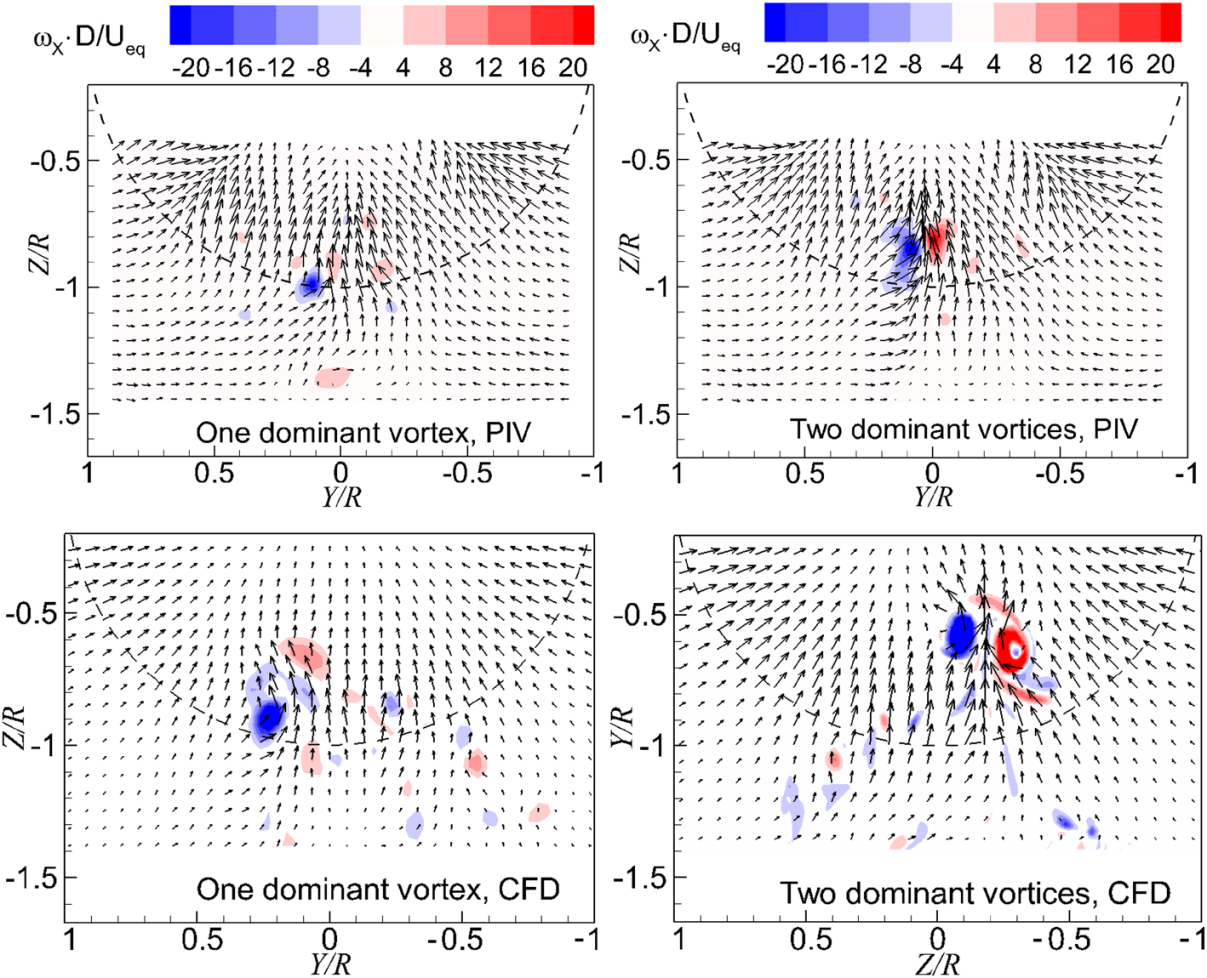
Instantaneous flow fields in the wall-normal plane. *Left column* one dominant vortex; *right column* two dominant vortices. $T_{\text{c}}$ = 27.3*, h/R* = 1.46

The instantaneous flow fields in the wall-normal plane just upstream of the propeller ($X = - 0.08 R$, 12 mm upstream of the disk model as defined in Fig. 4) are presented in Fig. 9, where the colour-coded contour of the axial vorticity component is superimposed on the velocity vector field. The vortices entering the propeller can be identified by the peak values of vorticity. There is one dominant vortex shown in the left column of Fig. 9, whereas there are two dominant vortices in the right column of Fig. 9.

**Fig. 10:**
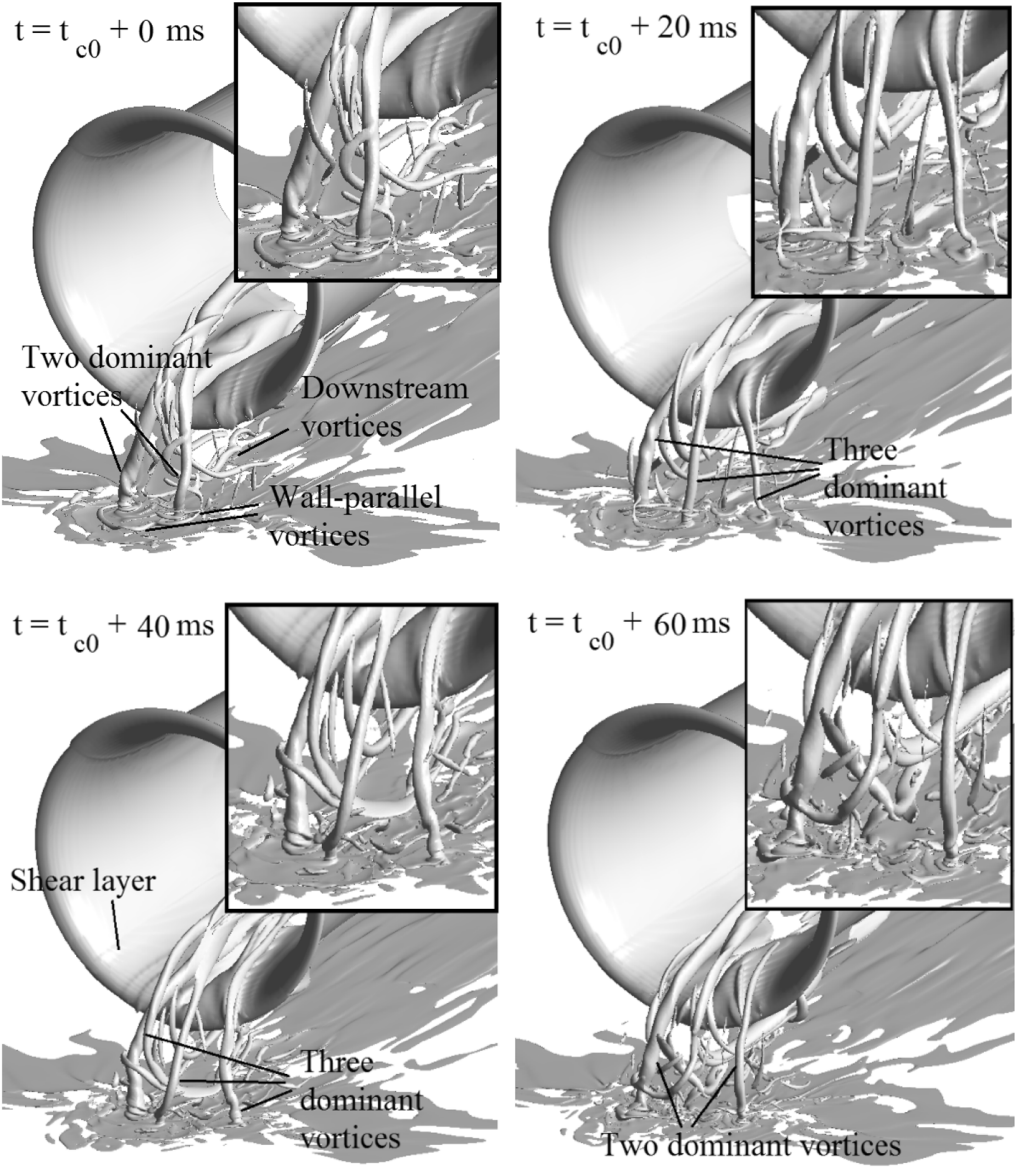
Three-dimensional flow fields in a sequence of time, represented by iso-surface of vorticity magnitude of $\left| \omega \right| \cdot D/U_{\text{eq}} = 17$. $T_{c} = 27.3$, $h/R = 1.46$

Based on the flow fields in the planes both near the ground and upstream of the propeller, the CFD results exhibit good agreement with the PIV measurement results. This implies that the flow field induced by the actuator disk model can represent that induced by a propeller. Therefore, the three dimensional flow fields represented by displaying the iso-surface of a vorticity magnitude are shown in Fig. 10, and it can be considered as representing the propeller induced flow.

A sequence of example flow fields in 60 ms and the time interval of 20 ms are chosen to explain a typical process of the flow development, as shown in Fig. 10. The flow field at the selected starting instant, $t = t_{\text{co}} + 0\,{\text{ms}}$, features a pair of dominant vortices. $t_{\text{co}}$ is selected at a random instant of the flow. It is also noted that there are a set of concentrated vortices transported from the downstream field and sucked into the propeller plane. Furthermore, there are wall-parallel vortices surrounding the dominant wall-normal vortices. At the subsequent time, $t = t_{\text{co}} + 20 \,\,{\text{ms}}$, one noticeable change is that another dominant vortex is formed on the right-hand side of the figure. This new vortex becomes relatively stronger at the time instant $t = t_{\text{co}} + 40 \,\,{\text{ms}}$. At the same time, this vortex in the middle of the figure moves toward the left vortex. At $t = t_{\text{co}} + 60 \,\,{\text{ms}}$, the foot of the middle vortex surrounds the left vortex, and only two dominant vortices exist in the flow field again. In addition, the vortex tube downstream of the actuator disk is observed, which is due to the shear between the slipstream of the propeller and the outer flow field.

## Vorticity source of ground vortices

### Lateral component of vorticity generated by pressure gradient on the ground

After the description of the occurrence of ground vortices by CFD simulations, one further step taken is to investigate the vorticity source of these vortices. As derived by Lighthill and reported in Lighthill (1963), the vorticity generation equation on a steady wall is:

5 $$\nu \left( {\frac{{\partial \omega_{X} }}{\partial Z}\vec{i} + \frac{{\partial \omega_{Y} }}{\partial Z}\vec{j}} \right) = \vec{k} \times \frac{1}{\rho }\nabla p_{Z = 0} .$$

$\nu$ is the kinematic viscosity; $\omega$ is vorticity; $\vec{i},\vec{j},\vec{k}$ are unit vectors in $X,Y,Z$ directions, respectively; $p_{Z = 0}$ is the static pressure on the ground. The terms $\nu \frac{{\partial \omega_{X} }}{\partial Z}$ and $\nu \frac{{\partial \omega_{Y} }}{\partial Z}$ represent the ‘total vorticity flux out of the solid surface per unit area per unit time’ (Lighthill 1963).

It shows that the production of the wall-parallel component of vorticity is due to the pressure gradient on the wall. CFD results from the simulations of the actuator disk model are analysed for the vorticity source entering ground vortices, which are performed at the condition of $T_{\text{c}}$ = 27.3*, h/R* = 1.46. The component of vorticity in the lateral direction (*Y* direction) in the domain close to the wall is analysed in this section in the symmetry plane $Y = 0$ as defined in Fig. 3.

One instantaneous flow field is investigated to analyse the vorticity source on the ground. This flow field corresponds to the one which was displayed in the top left of Fig. 10. The mechanism found in this instant can also be applied to other instants, because each instantaneous flow is a solution of Navier–Stokes equations. In addition, the flow field is highly unsteady and the analysis on the time-averaged result would not give a comprehensive explanation of the vorticity source, so an instantaneous flow field is analysed.

**Fig. 11:**
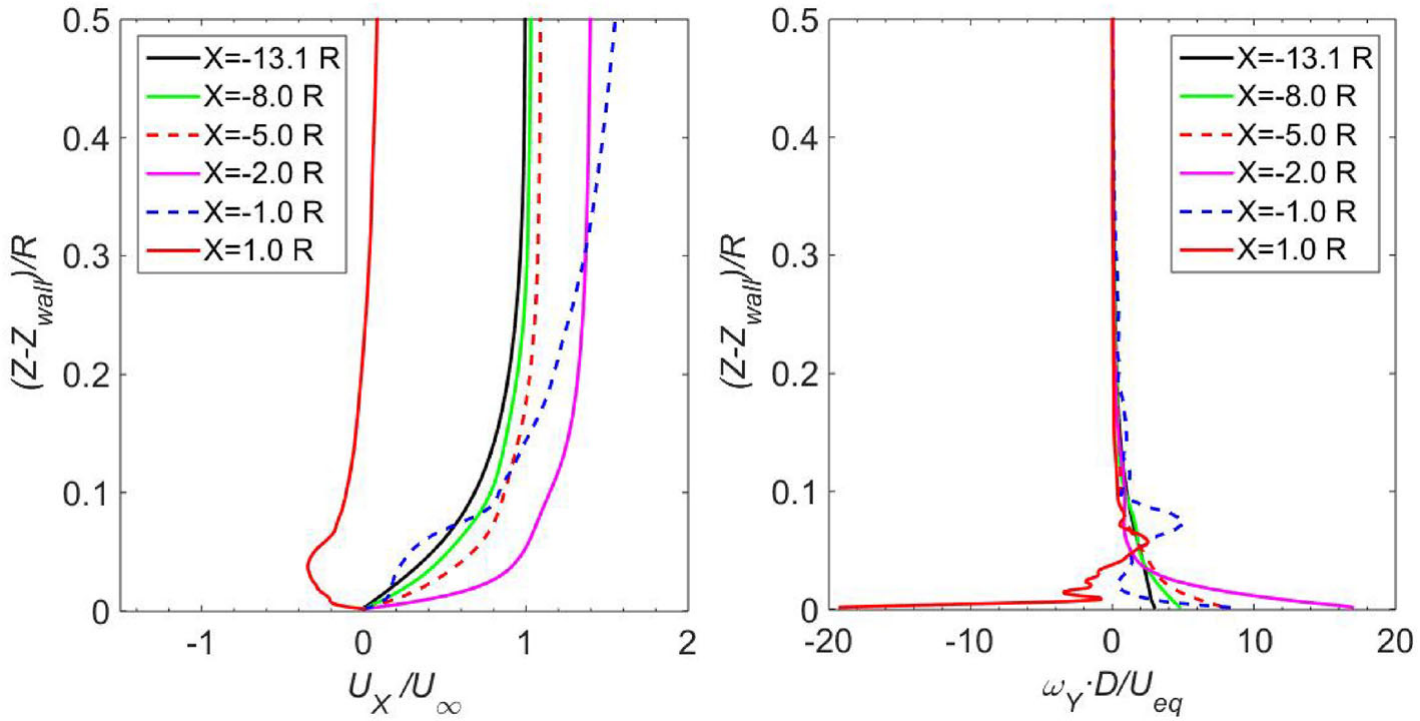
*Left* profile of the *X*-component velocity in the boundary layer; *right* distribution of the *Y*-component vorticity in the boundary layer. The data are extracted from the plane $Y = 0$

The wall-normal distribution of the normalized streamwise velocity in the plane of $Y = 0$ is shown in the left-hand side of Fig. 11. At the position of $X = - 13.1 R$ (velocity inlet), the velocity profile resembles the prescribed profile at the velocity inlet. The boundary layer has a thickness of $0.45 R$ at the velocity inlet. As the flow progresses downstream, the *X*-component of the velocity has a trend of increasing both inside and outside the boundary layer, and the velocity outside the boundary layer reaches $1.3 U_{\infty }$ at the position of $X = - 2.0 R$. At the position of $X = - 1.0 R$, the flow field becomes three-dimensional and the *X*-component of the velocity changes noticeably in the wall-normal direction. At the position of $X = 1.0 R$, adverse flow with a negative value of the *X*-component velocity is observed near the wall.

The distribution of vorticity close to the wall in the symmetry plane $Y = 0$ is shown in the right-hand side of Fig. 11. At positions from $X = - 13.1 R$ to $X = - 2 R$, the magnitude of vorticity on the wall increases monotonically from $\omega_{Y} \cdot D/U_{\text{eq}} = 3.0$ to $16.9$. In other words, the vorticity at the wall at the position $X = - 2 R$ is 5 times as large as that at $X = - 13.1 R$. Downstream of the propeller, e.g. $X = 1 R$ in the current analysis, the magnitude of the vorticity on the ground is $|\omega_{Y} | \cdot D/U_{\text{eq}} = 19.2$, which is more than 6 times as large as that at $X = - 13.1 R$. It is also noted that the vorticity distribution at $X = 1 R$ is not smooth near the ground, which is probably due to the complex vortex structures in this region as shown in the top left figure of Fig. 10.

**Fig. 12:**
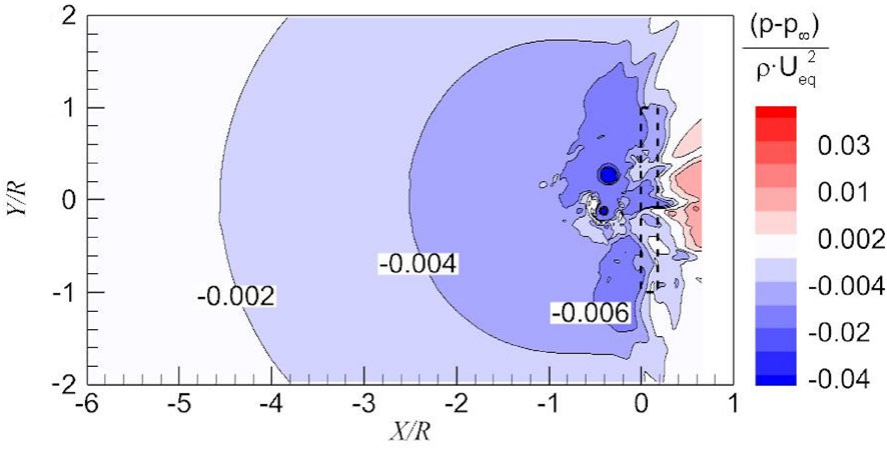
Pressure distribution on part of the ground

The cause for the increment of vorticity magnitude upstream of the propeller and the negative vorticity downstream of the propeller is investigated by analysing the pressure distribution on the ground. This pressure distribution is shown for the upstream and downstream vicinity of the propeller in Fig. 12. Generally, the pressure decreases upstream of the propeller and increases downstream of the propeller in the streamwise direction, and the pressure decreases toward the propeller in the lateral direction. The two local pressure minima occur at the centres of the dominant vortices, which are due to the centrifugal force of the swirling flow.

The production rate of the *Y*-component vorticity is obtained from Eq. (5) as:

6 $$\nu \frac{{\partial \omega_{Y} }}{\partial Z} = (\partial p/\partial X)/\rho .$$

**Fig. 13:**
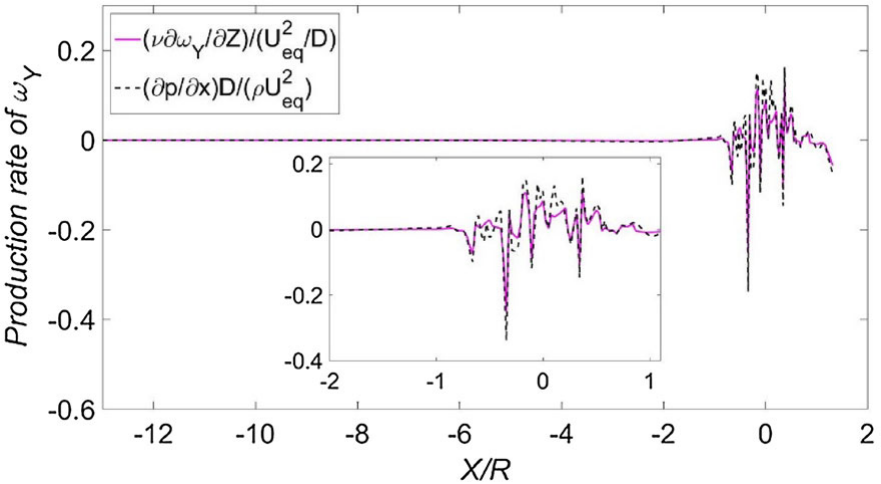
Terms of the vorticity production equation. The small window shows a zoom-in section. Data are extracted from the intersection line between the symmetry plane and the wall

To use this equation to verify the numerical results, a comparison between the terms on the left- and right-hand sides of Eq. (6) is shown in Fig. 13, which is extracted from the symmetry line on the ground. The two terms agree with each other very well. In the region close to the actuator disk, discrepancies are observed between the two terms, which is due to the *Y* component of the vorticity being depleted in feeding the other components (e.g. the wall-parallel component of vorticity is turned into the wall-normal component). However, as found in Fig. 13, the trends of the two terms are consistent in most of the domain.

**Fig. 14:**
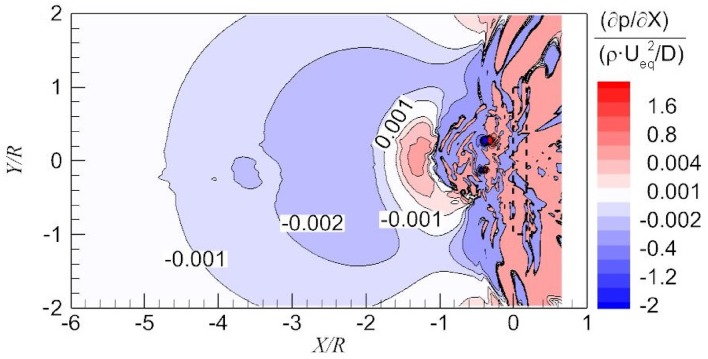
Production rate of the *Y*-component vorticity from pressure gradient in the longitudinal component along the wall

After the validation of the vorticity source on a line, the production of the *Y*-component vorticity induced by the pressure gradient on part of the ground plane is shown in Fig. 14. Far upstream of the actuator disk, the pressure gradient (vorticity production rate) is nearly zero. There is a negative pressure gradient on the wall from $X = - 6 R$ to $X = - 2 R$ as shown in Fig. 14. Consequently, *Y*-component vorticity is produced. This locally generated vorticity is then diffused into the far field, and finally the vorticity in the whole boundary layer increases as shown in the right-hand side of Fig. 11.

Downstream of the propeller, the pressure gradient is positive. Initially, the vorticity convected from the far field boundary layer still dominates. Due to the positive vorticity gradient induced by the actuator disk, the vorticity on the wall keeps decreasing and finally becomes negative. In other words, due to the low-pressure region upstream of the propeller as shown in Fig. 12, there is a reverse flow downstream of the propeller. Vorticity of negative sign is involved in this reverse flow due to the pressure gradient.

### Streamwise component of vorticity generated by pressure gradient on the ground

**Fig. 15:**
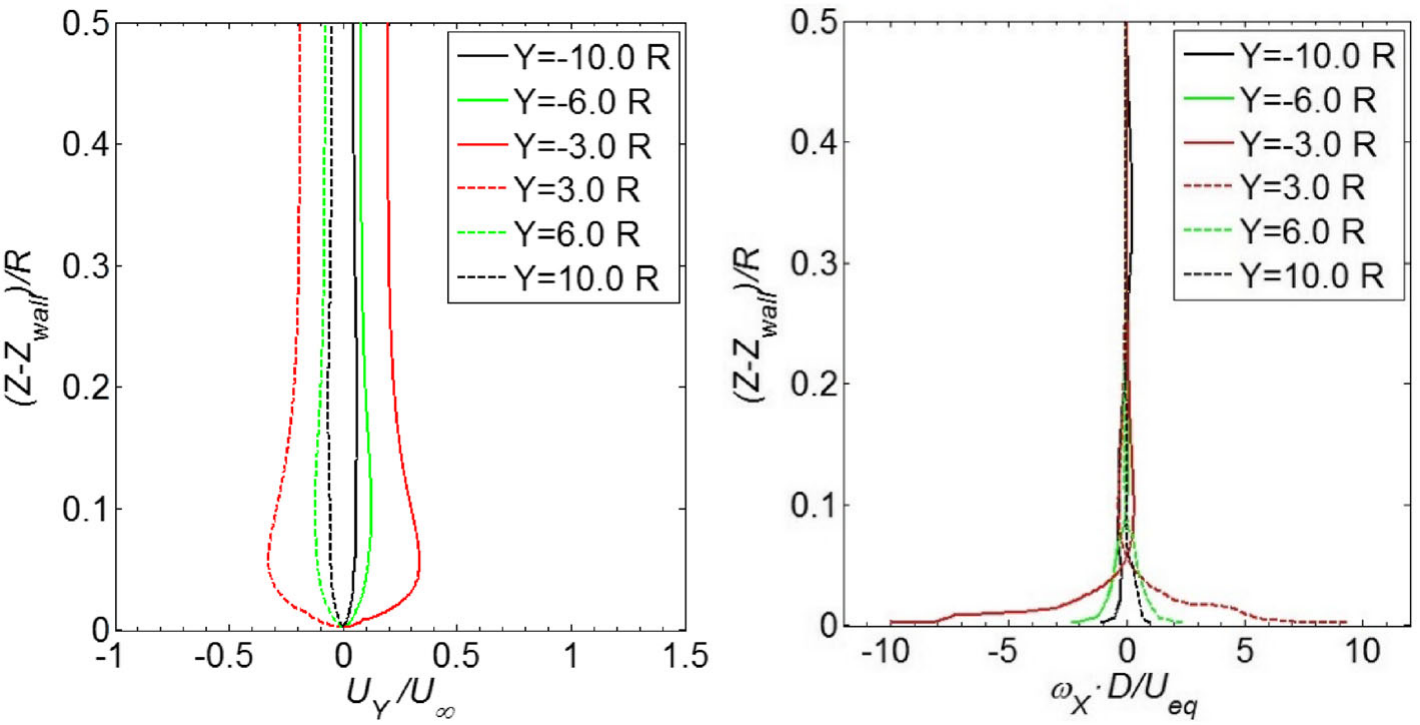
*Left* profile of the *Y*-component velocity in the boundary layer; *right* distribution of the *X*-component vorticity in the boundary layer. The data are extracted from the plane of $X = - 1R$

Following the same method as above, the flow field in the plane $X = - 1.0 R$ (as shown in Fig. 3) is analysed for the production rate of the *X*-component of the vorticity. First, the distribution of the *Y*-component velocity in this plane is plotted in the left-hand side of Fig. 15. For positions far from the centre axis, e.g. $Y = \pm 10.0 R$, the magnitude of the *Y*-component velocity is approximately zero. At the positions close to the propeller, e.g. $Y = \pm 6 R$ and $\pm 3 R$, the magnitude of the *Y*-component velocity increases due to the propeller suction effect.

The distributions of the *X*-component vorticity are presented in the right-hand side of Fig. 15. Vorticity is nearly zero for the positions far away from the centre axis, e.g. at $Y = \pm 10 R$. At $Y = \pm 6 R$, the magnitude of vorticity on the wall increases to $\omega_{X} \cdot D/U_{\text{eq}} = 2.5$. At positions of $Y = \pm 3 R$, the vorticity magnitude further increases to $\omega_{X} \cdot D/U_{\text{eq}} = 10.0$. The magnitudes of the *X*-component vorticity at the wall at the positions $Y = \pm 3 R$ are more than 3 times as large as the magnitude of the *Y*-component vorticity at the wall in the far field boundary layer, as shown by the black curve in the right-hand side of Fig. 11.

### Development of the wall-normal component of the vorticity

As elaborated in (Lighthill 1963), vorticity production in the wall-normal component is due to the divergence of vorticity on the ground. The equation of the vorticity production rate in the wall-normal component is

7 $$\nu \frac{{\partial \omega_{Z} }}{\partial Z} = - \nu \left( {\frac{{\partial \omega_{X} }}{\partial X} + \frac{{\partial \omega_{Y} }}{\partial Y}} \right).$$

**Fig. 16:**
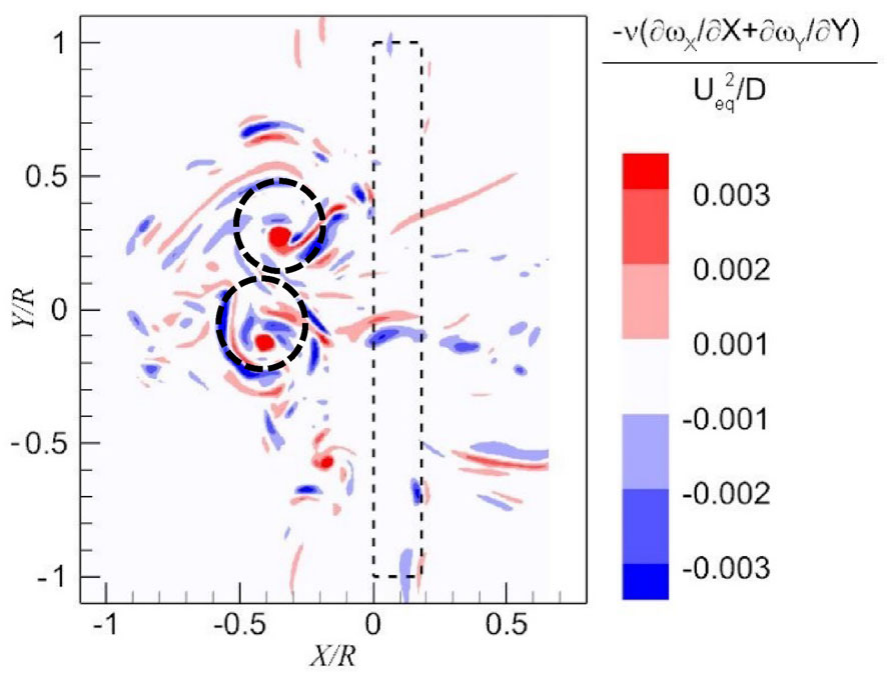
Divergence of the wall-parallel components of the vorticity vector on the wall, $- \nu \left( {\frac{{\partial \omega_{X} }}{\partial X} + \frac{{\partial \omega_{Y} }}{\partial Y}} \right)/(U_{\text{eq}}^{2} /D)$

The term on the right-hand side of Eq. (7) is calculated in the plane 7 mm above the ground, and it is shown in Fig. 16. Strong divergence is observed in the vortex core region as marked by the dashed black circles. These regions with strong divergence coincide with the positions of dominant vortices as shown in the top left of Fig. 10. It is also noted that the divergence with non-zero values covers a wide area, which means the wall-normal component of the vorticity is distributed.

## Domain boundary of ground vortex occurrence

**Fig. 17:**
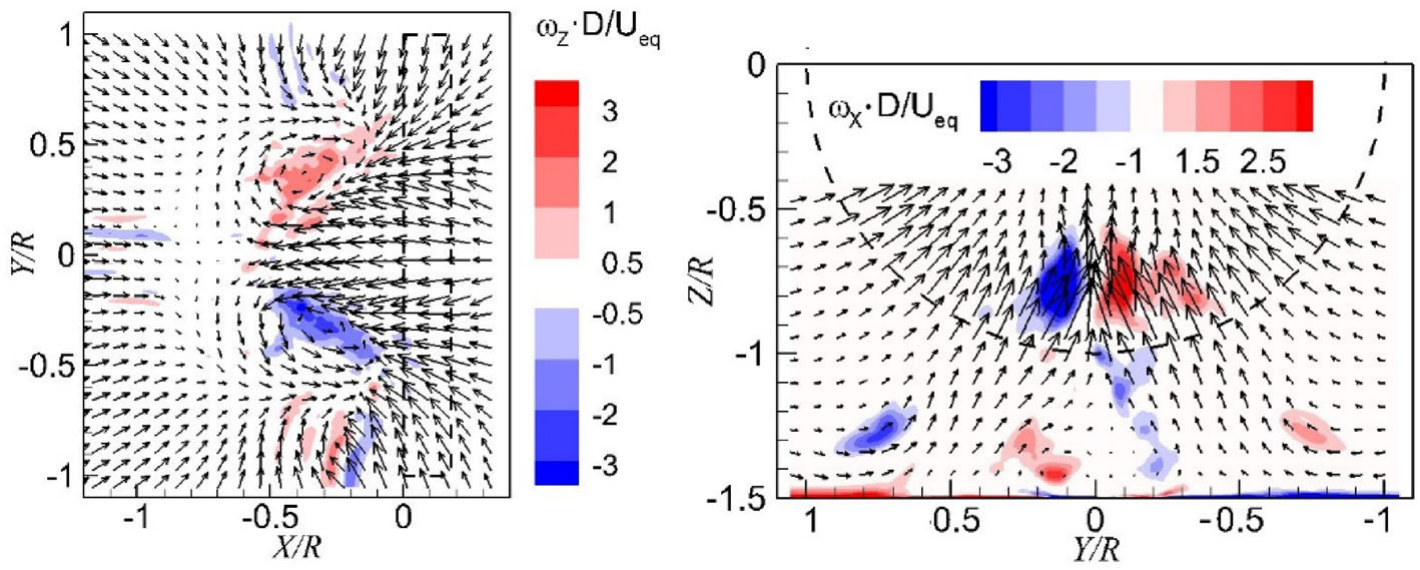
Distributions of vorticity in the time-averaged flow field (CFD results). *Left* wall-parallel plane; *right* wall-normal plane directly upstream of the propeller. $T_{\text{c}}$ = 27.3*, h/R* = 1.46

**Fig. 18:**
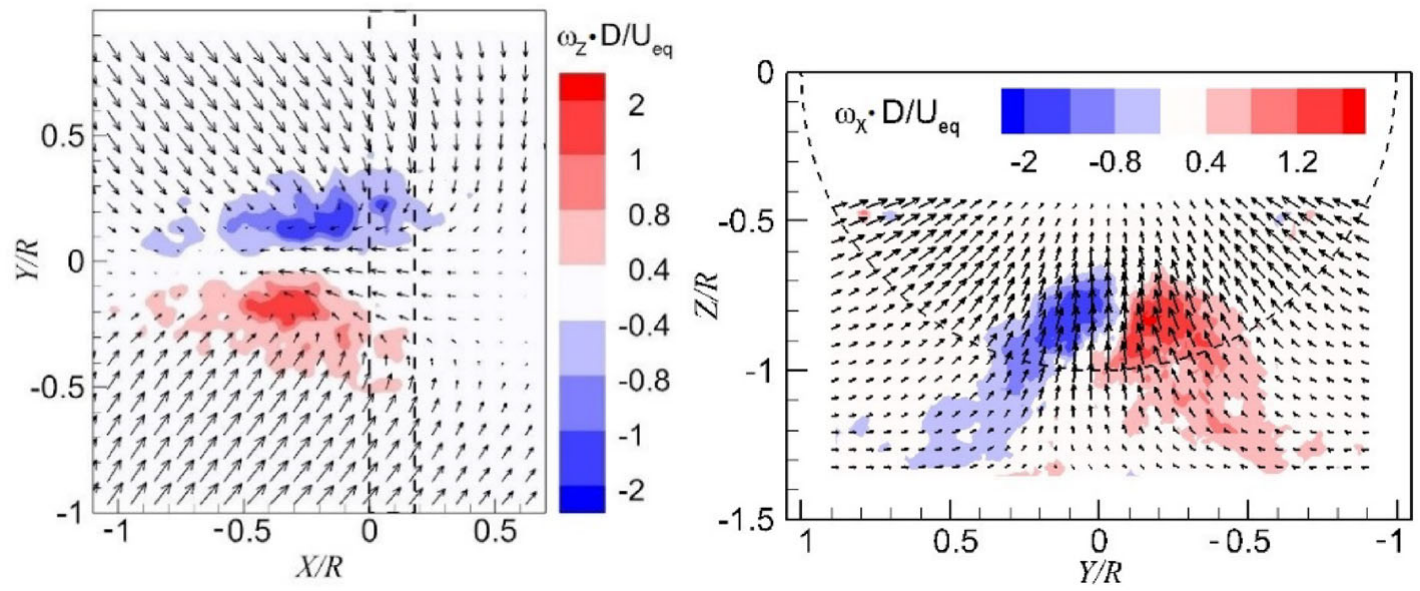
Distributions of vorticity in the time-averaged flow field (experimental results). *Left* wall-parallel plane; right: wall-normal plane directly upstream of the propeller. $T_{\text{c}}$ = 27.3, *h/R* = 1.46

An important application of the current numerical method is to predict the occurrence of ground vortices. The determination method for the occurrence of vortices is to detect regions of concentrated vorticity in the time-averaged flow field, as well as by the visualization of the flow field. The concentrated vorticity in the wall-parallel plane and the wall-normal plane are shown in the left- and right-hand sides of Fig. 17, respectively, from CFD results. The results obtained from experiments are shown in Fig. 18. The time-averaged flow fields show a pair of vortices and the results from the two methods are consistent but exhibit some discrepancies. The CFD results show a more concentrated region of vorticity than the experimental results, which is ascribed to the impact of the unsteadiness in the free stream, blades rotation, and slipstream of the propeller in the experiments.

A parameter is defined here to evaluate the concentration of vorticity, which is the ratio between the maximum magnitude of vorticity in the time-averaged flow field and the value in the region assumed to be unaffected by the ground vortices. These unaffected regions are chosen at a $3 \times 3$ kernel centred at $[X/R, Y/R] = [ - 1, 0.7]$ in the wall-parallel plane, and $[Y/R,Z/R] = [ - 0.8, - 1]$ in the wall-normal plane. The criterion applied in the paper is that if the ratio is larger than 10 (this value is determined by considering the concentrated vorticity should be one order of magnitude larger than the vorticity from turbulence), it is considered to be concentrated vorticity; otherwise there is no concentrated vorticity in the flow field. It should be noted that this criterion is only valid if the fluctuation of the vorticity field is moderate. Large fluctuations would also decrease the mean vorticity field and prevent detection of vortices with this criterion.

**Fig. 19:**
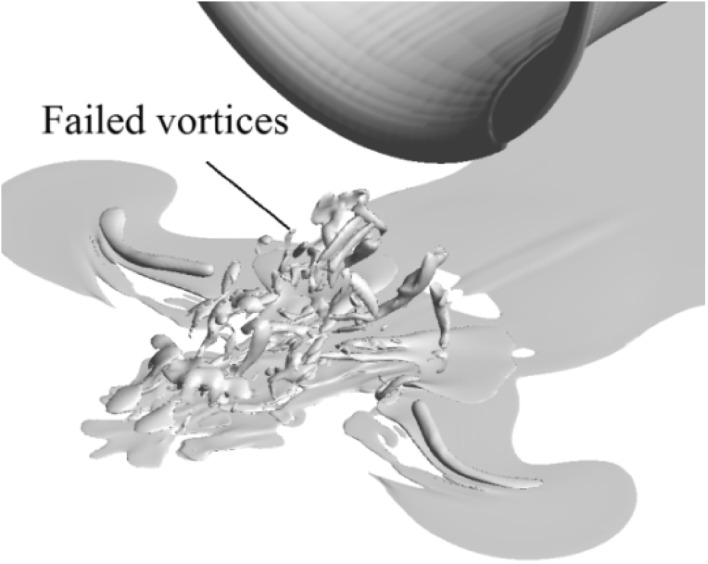
Three-dimensional flow field with failed vortices, $\left| \omega \right| \cdot D/U_{\text{eq}} = 8$. $T_{\text{c}} = 14$, $h/R = 1.85.$

**Fig. 20:**
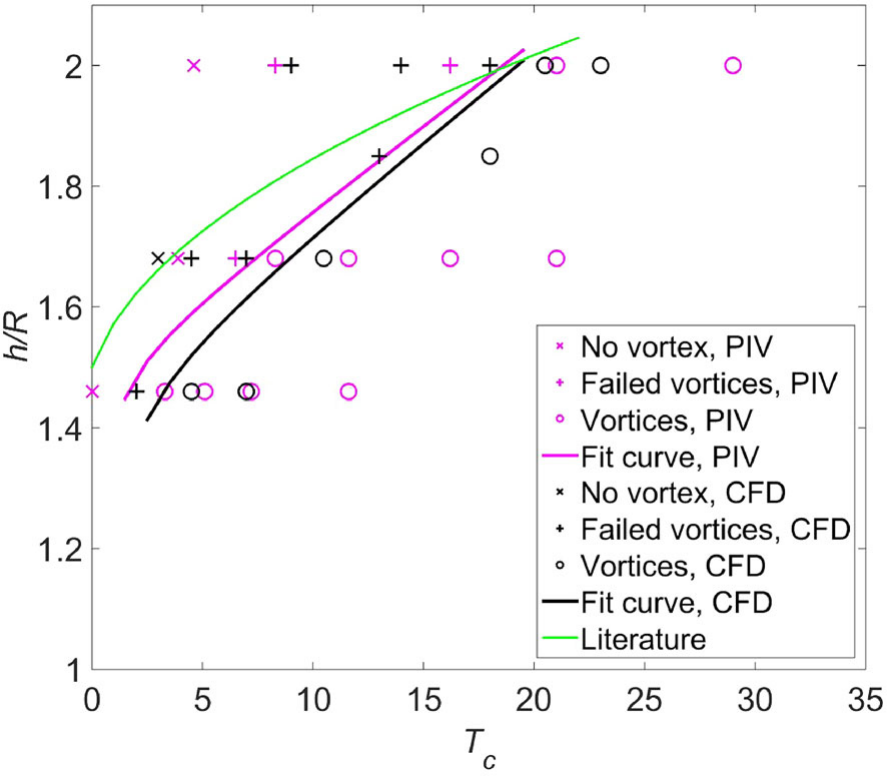
Domain boundary of occurrence of ground vortices induced by the propeller. The data from literature (*green curve*) are reported in Nakayama and Jones (1996)

If there is no concentrated vorticity in the flow field either near the ground or upstream of the propeller, it is defined as the case ‘no vortex’. If there is concentrated vorticity in the flow field both near the ground and upstream of the propeller, it is defined as the case ‘vortices entering the propeller (vortices)’. If there is concentrated vorticity in the flow field near the ground but not existing directly upstream of the propeller, it is defined as the case ‘failed vortices’ (a three dimensional flow representing this type of flow is shown in Fig. 19). To build a map of vortex states at different thrust coefficients and height ratios, cases by tuning these two parameters are tested. A map of ‘no vortex’ (symbol ‘×’), ‘failed vortices’ (symbol ‘+’) and ‘vortices entering propeller’ (symbol ‘o’) from both the PIV results and CFD simulations is shown in Fig. 20. The *X*-axis of the plot is the thrust coefficient $T_{\text{c}}$, and the *Y-*axis is the height ratio, $h/R$.

The boundary curves by connecting the midpoints between the vortices and failed vortices at $h/R = 1.46, 1.7,$ 1.85 and 2.0 are shown by the solid purple and black curves in Fig. 20. The boundary curves divide the domain into two sub-domains: the upper left domain represents no vortex entering the propeller, while the bottom right domain represents vortices entering the propeller. In other words, as the height ratio decreases and the thrust coefficient increases, the ground vortices occur.

As found in Fig. 20, both the boundary curves from PIV results and CFD results agree quite well. Because the number of blades is assumed to be infinite, and the loading in the radial direction is assumed to be constant for the actuator disk model, the consistency with the experimental results implies that the occurrence of ground vortices is not sensitive to these parameters and it is mainly determined by the total thrust generated by the propeller.

It should also be noted that only the parameters of $T_{\text{c}}$ and $h/R$ are taken into account when predicting the occurrence of ground vortices, but the parameter of the free stream velocity is kept constant at 2.7 m/s. The vorticity transported from the free stream velocity is also one source of vorticity to form ground vortices as discussed before. The free stream velocity is very likely one factor determining the occurrence of ground vortices as well. Although different free stream velocities are not investigated in the current research, the contribution of the free stream in the formation of ground vortices was already discussed in Sect. 4.

In addition, the green curve is the domain boundary of occurrence of ground vortices for turbofans which is reported in Nakayama and Jones (1996). It shows that the ground vortices induced by turbofans occur at lower thrust coefficients than that induced by the propeller for the same height ratio. This discrepancy is perhaps due to the different free stream velocities and fluctuating characteristics, which play a role in the formation of the ground vortices (Brix et al. 2000). Generally, these tests with turbofans were conducted at larger free stream velocities than our results, which mean the vorticity in the far field boundary layer is larger. Therefore, the ground vortices can occur at lower thrust coefficients.

## Conclusions

By employing the actuator disk model, the flow due to the interaction between the propeller and the ground is investigated. A system of ground vortices ascending from the ground toward the propeller is observed. The flow fields from the CFD simulations are compared with the experimental results in different planes around the rotor which the ground vortices go through. Good agreement is found from these two datasets.

According to literature, the vorticity source of ground vortices mainly originates from the free stream velocity at the headwind condition. However, Lighthill’s vorticity generation equation clearly showed that the vorticity could be generated when there is a pressure gradient on the wall. By inspecting the pressure gradient on the wall and the vorticity generation rate, a connection is found between the two terms in our numerical results. Therefore, this research explicitly presents a second source of vorticity, which is attributed from the propeller suction.

The verified numerical results are also utilized to describe the conditions under which ground vortices occur. As the elevation of the propeller from the ground decreases, and the propeller thrust coefficient increases, the ground vortices occur. A boundary curve of ground vortex occurrence is also built, which is based on discrete test points. Both the boundary curves from the PIV results and the CFD results agree quite well. The vortices failing to reach the propeller are first visualized in three-dimensional flow by our results, which were also proposed to exist in PIV results deduced from discrete measurement planes (Wang and Gursul 2012).
